# *Notes from the Field:* Monkey Bite in a Public Park and Possible Exposure to Herpes B Virus — Thailand, 2018

**DOI:** 10.15585/mmwr.mm6909a6

**Published:** 2020-03-06

**Authors:** Alexander C. Wu, Steven I. Rekant, Elizabeth R. Baca, Renee M. Jenkins, Ludmila M. Perelygina, Julia K. Hilliard, D. Scott Schmid, Richard F. Leman

**Affiliations:** ^1^Epidemic Intelligence Service, CDC; ^2^Northwest Portland Area Indian Health Board, Portland, Oregon; ^3^Acute and Communicable Disease Prevention, Oregon Public Health Division, Portland, Oregon; ^4^Clackamas County Public Health Division, Oregon City, Oregon; ^5^Division of Viral Diseases, National Center for Immunization and Respiratory Diseases, CDC; ^6^National B Virus Resource Center Laboratory, Georgia State University, Atlanta, Georgia.

On January 7, 2019, the Oregon Public Health Division (OPHD) was contacted by a local health department regarding an Oregon teen who, on December 24, 2018, was bitten by a macaque monkey (Figure) in a public park in Phuket, Thailand. The bleeding wound was immediately rinsed with bottled water without soap. Subsequently, hotel staff members applied a topical pain reliever. The following day, the teen went to a local clinic in Thailand and received the first dose of rabies postexposure prophylaxis vaccine; rabies immune globulin was not administered. She received 2 additional doses of rabies vaccine while in Thailand.

**FIGURE:**
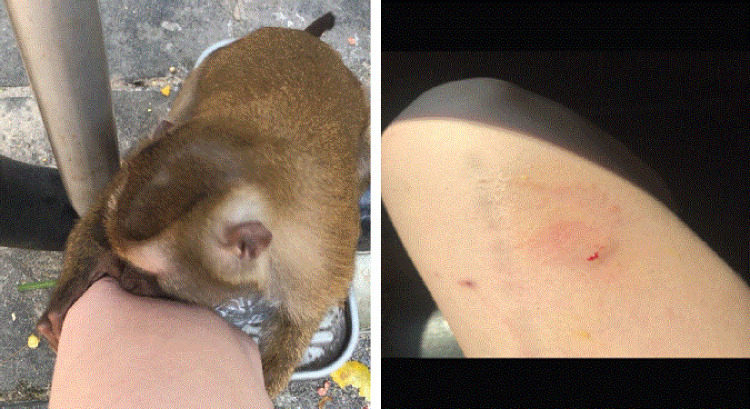
Macaque monkey biting an Oregon resident in a public park in Thailand and the resultant wound — 2018 Photo/patient

On January 5, 2019, the patient left Thailand and was evaluated by a physician in Oregon on January 7. The physician contacted the local health department, seeking guidance about when to administer the final dose of rabies vaccine. Upon learning about the macaque bite, the local health department contacted OPHD, where staff members expressed concern about possible exposure to *Macacine herpesvirus* 1 (B virus). This virus, commonly found in macaques,* can, in rare cases, cause severe encephalitic infection in humans if not treated promptly (*1*). The case fatality rate of untreated B virus infection approaches 80% (*2*). OPHD contacted CDC, and the National B Virus Resource Center (NBVRC) in Atlanta, Georgia, to discuss testing.^†^

OPHD recommended that if illness compatible with B virus infection developed (e.g., fever, chills, myalgia, headache, blisters or discomfort near the wound, or problems with coordination) the patient should seek medical evaluation, and the provider should notify NBVRC immediately. On January 8, 2019, the patient received the final dose of rabies vaccine. Per recommendations for persons possibly exposed to B virus, serum specimens were collected at that visit and 3 weeks later^§^ (January 29) for B virus immunoglobulin (Ig) M and IgG testing at NBVRC.^¶^ Neither specimen was positive for antibodies against B virus.

Following an initial exposure to B virus, the peripheral viral load can be insufficient to stimulate an immune response and can result in negative tests for antibodies against B virus. B virus can migrate to the dorsal ganglion and cause infection years later (Julia Hilliard, NBVRC, personal communication, 2019). Because B virus can establish a lifelong latent infection with possible subsequent illness (*3*), the patient was advised always to carry a Medical Alert card** in case symptoms occur despite her initial negative tests (*4*).

Symptomatic B virus infection in humans is rare. Seroconversion in some persons suggests that asymptomatic infection can occur (Julia K. Hilliard, NBVRC, personal communication, 2019). Nearly all documented B virus infections in humans involved exposures in laboratories or animal facilities (*4*). Transmission from macaques to humans in public settings, such as parks, has not been documented. Nonetheless, macaques in these settings often carry B virus and can shed the virus asymptomatically (*4*); the macaque in this case ran away and could not be tested. Although the risk for human B virus disease from macaque exposure in these settings is considered low, precautions are indicated given the severe consequences of infection. Macaque bites and scratches are of particular concern (*1*,*4*). Wounds from macaque bites should be scrubbed with soap, detergent, or iodine for 15 minutes and irrigated with running water for an additional 15–20 minutes before seeking medical attention.^††^ Treatment varies based on the details of the incident.^§§^ There is no vaccine against B virus.

Rabies from nonhuman primate bites is uncommon because primates are not primary rabies reservoirs. Nonetheless, rabies postexposure prophylaxis for victims of nonhuman primate bites in countries where rabies is enzootic should be considered (*5*). Persons visiting areas with free-ranging macaques should avoid close contact with these animals (*1*). Macaque bites or scratches should be thoroughly washed, and medical treatment should be sought immediately.

## Footnotes

*
https://www.cdc.gov/herpesbvirus/cause.html .

†
https://www.cdc.gov/herpesbvirus/laboratory.html .

§
https://www.cdc.gov/herpesbvirus/laboratory.html .

¶ http://biotech.gsu.edu/virology/PDFs/2012%20Sample%20Collect%20&%20Shipmt.pdf.

** http://biotech.gsu.edu/virology/PDFs/2011%20Medical%20Alert.pdf.

††
https://www.cdc.gov/herpesbvirus/firstaid-treatment.html .

§§ https://www.cdc.gov/herpesbvirus/healthcare-providers.html.
